# Animal sales from Wuhan wet markets immediately prior to the COVID-19 pandemic

**DOI:** 10.1038/s41598-021-91470-2

**Published:** 2021-06-07

**Authors:** Xiao Xiao, Chris Newman, Christina D. Buesching, David W. Macdonald, Zhao-Min Zhou

**Affiliations:** 1 Key Laboratory of Southwest China Wildlife Resources Conservation (Ministry of Education), China West Normal University, Nanchong, 637009 People’s Republic of China; 2 Lab Animal Research Center, Hubei University of Chinese Medicine, Wuhan, People’s Republic of China; 3 Wildlife Conservation Research Unit, Department of Zoology, The Recanati-Kaplan Centre, University of Oxford, Oxford, UK; 4 Cook’s Lake Farming, Forestry and Wildlife Inc. (Ecological Consultancy), Queens County, NS Canada; 5 Department of Biology, The University of British Columbia/ Okanagan, Kelowna, BC Canada; 6 Key Laboratory of Environmental Science and Biodiversity Conservation (Sichuan Province), China West Normal University, Nanchong, People’s Republic of China

**Keywords:** Socioeconomic scenarios, Conservation biology, Viral infection

## Abstract

Here we document 47,381 individuals from 38 species, including 31 protected species sold between May 2017 and November 2019 in Wuhan’s markets. We note that no pangolins (or bats) were traded, supporting reformed opinion that pangolins were not likely the spillover host at the source of the current coronavirus (COVID-19) pandemic. While we caution against the misattribution of COVID-19’s origins, the wild animals on sale in Wuhan suffered poor welfare and hygiene conditions and we detail a range of other zoonotic infections they can potentially vector. Nevertheless, in a precautionary response to COVID-19, China’s Ministries temporarily banned all wildlife trade on 26th Jan 2020 until the COVID-19 pandemic concludes, and permanently banned eating and trading terrestrial wild (non-livestock) animals for food on 24th Feb 2020. These interventions, intended to protect human health, redress previous trading and enforcement inconsistencies, and will have collateral benefits for global biodiversity conservation and animal welfare.

## Introduction

Alongside extensive research into the epidemiology, virology and medical treatment of SARS-CoV-2, known generally as COVID-19, it is also vital to better understand and mitigate any role that may have been played by the illegal wildlife trade (IWT) in China, in initiating this pandemic^1^. COVID-19 was first observed when cases of unexplained pneumonia were noted in the city of Wuhan, Hubei Province, in late 2019^2^. Like the SARS-CoV epidemic (another coronavirus, for which there is still no cure) that began in Guangdong Province in 2002^3^, this latest coronavirus most closely resembles types found in bats^4^. Initial media coverage suggesting that COVID-19 may have spilled-over via pangolins has been refuted^5, 6^; probably pangolins are simply a natural reservoir of SARS-CoV-2^7–9^ along with palm civets (*Paguma larvata*)^10^.

The World Health Organization (WHO) sent an investigative team to Wuhan, from 14 January–10 February 2021, to try to retrospectively ascertain what wildlife was being sold in local wet markets in this region^1^. Their findings were inconclusive, with markets having been closed down completely at that point for 4 months; however, they did recommend that pangolins should be included in the search for possible natural hosts or intermediate hosts of the novel coronaviruses^1^.

Here we present a unique and original dataset recording wild animal sales across Wuhan City’s animal markets between May 2017 and November 2019. We investigate which wildlife species (including both wild-caught and farmed non-domesticated species) were actually being sold for food and as pets, what quantities were involved, and to what extent vendors violated their trading permits. We also list zoonotic pathogens recorded in Chinese wild animal markets and/ or farms since 2009, along with broader notes on infections established for these species. We evaluate these data in the context of China’s renewed commitment to enforce and build on pre-existing laws within a culture of traditional wildlife exploitation. Finally, we make pragmatic policy recommendations for better regulating the animal trade pervasive in China, integrating with ethics, education and enforcement.

## Materials and methods

**Figure 1:**
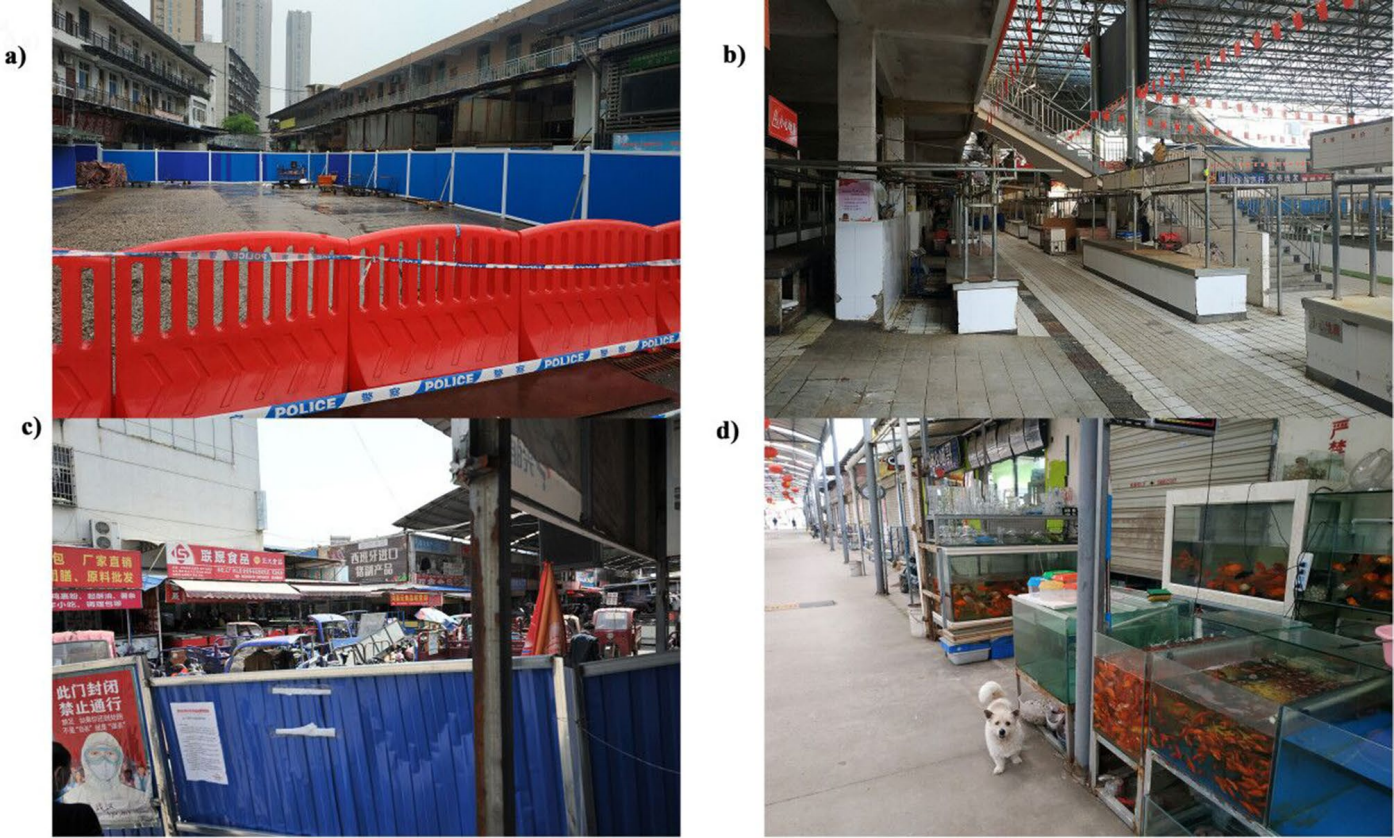
(**a**) Huanan Seafood market, (**b**) Qiyimen live animal market, (**c**) Baishazhou market and (**d**) Dijiao outdoor pet market (note stray dog) photographed on 10th April 2020.

Serendipitously, prior to the COVID-19 outbreak, over the period May 2017–Nov 2019, we were conducting unrelated routine monthly surveys of all 17 wet market shops selling live wild animals for food and pets across Wuhan City (surveys were conducted by author X.X.). This was intended to identify the source of the tick-borne (no human-to-human transmission) Severe Fever with Thrombocytopenia Syndrome (SFTS), following an outbreak in Hubei Province in 2009–2010 in which there was an unusually high initial case fatality rate of 30%^11^. These shops selling live, often wild, animals included two at Baishazhou market (a large market comprising c. 400 other types of shop), seven at Huanan seafood market (c. 120 other shops), four at Dijiao outdoor pet market (c. 100 other shops), and four at Qiyimen live animal market (c. 40 other shops). Other shops sold a variety of goods, such as live and butchered livestock and poultry, dairy produce, fish, shellfish, other food- related products and domesticated pets (Fig. 1 shows the appearance of these markets upon reopening on 8th April 2020).

As an objective observer unconnected to law enforcement X.X. was granted unique and complete access to trading practices. On each visit, vendors were asked what species they had sold over the preceding month and in what numbers, along with the prices (US$1:RMB¥6.759) and origin of these goods (wild caught or captive bred/ farmed). Additionally, to substantiate interview data, the number of individuals available for sale at the time of each visit was noted, and animals were checked for gunshot wounds (from homemade firearms—gun ownership is strictly regulated in China^12^) or leg-hold (snap) trap injuries, indicative of wild capture. For 15 species (3 mammals and all 12 reptiles) sold by weight, vendors did not record the number of individuals sold. In these instances, we report numbers of individuals observed to be on sale during monthly visits.

In China, wild animals are state property protected by the Wild Animal Conservation Law (WACL 1988, revised in 2004, 2009, 2016 and 2018), in concert with the Criminal Law (Article 341)^13^. Any convicted trader of species, and/or products derived thereof, protected by China’s list of Fauna under Special State Protection (LFSSP) and/or any non-native species listed in the Convention on International Trade in Endangered Species of Wild Fauna and Flora (CITES) Appendix I, II could face up to 15 years fix-term imprisonment accompanied by fines and/or the confiscation of property. Additionally, if any animal protected by China’s List of Terrestrial Wild Animals of Significant Ecological, Scientific, or Social Value Protected by the State (LESS) is taken from a wild population and traded for the purpose of food, the offender could face up to 3 years fix-term imprisonment accompanied by criminal detention, surveillance and/or fines. Correspondingly, any utilization of these protected species should be approved by the wild animal conservation and animal quarantine administrations through various regulatory schemes. We therefore also noted if vendors had necessary permits allowing them to sell livestock; specifically a License for Domestication and Breeding of Wild Animals, a License for Trade and Processing of Wild Animals and the quarantine certificate, which must be displayed to customers according to the WACL (2018), Animal Epidemic Prevention Law (2013) and Special Provisions of the State Council on Strengthening the Food Safety Supervision and Administration (2007).

All protocols in the market surveys were reviewed and approved by the Ethics Committee of Hubei University of Chinese Medicine (No. 20161111). All vendors provided informed written consent to participate in these surveys, and all protocols were performed in accordance with relevant guidelines and regulations.

## Results

### Animal sales from Wuhan’s markets

Across all 17 shops, vendors reported total sales of 36,295 individuals, belonging to 38 terrestrial wild animal species, averaging 1170.81 individuals per month (Standard deviation (SD) = 445.01, n = 31; Table 1). Including species sold by weight inflated this total to 47,381 individuals. Notably, no pangolin or bat species were among these animals for sale.

**Table 1:** List of 38 species sold in Wuhan City markets between May 2017–Nov 2019, including the mean number of live individuals sold per month and price (mean ± SD; n = survey rounds).

Species on sale	Monthly mean (and SD) number of individuals sold	Price (mean ± SD) $ per individual
**Mammals**		
Raccoon dog (*Nyctereutes procyonoides*)^W,R,F,†^	38.33 ± 17.24 (n = 30)	63.32 ± 15.46 (n = 5)
Amur hedgehog (*Erinaceus amurensis*)^R,F,†^	332.14 ± 190.62 (n = 28)	2.66 ± 0.41 (n = 5)
Siberian weasel (*Mustela sibirica*)^W,R,F,†^	(10.06 ± 12.09, n = 31)	11.24 ± 3.07 (n = 5)
Hog badger (*Arctonyx albogularis*)^W,R,F,†^	(6.81 ± 5.37, n = 31)	72.79 ± 34.08 (n = 5)
Asian badger (*Meles leucurus*)^W,R,F,†^	12.24 ± 7.39 (n = 29)	59.77 ± 15.89 (n = 5)
Chinese hare (*Lepus sinensis*)^W,R,F,†^	168.96 ± 89.06 (n = 29)	16.87 ± 2.88 (n = 5)
Pallas's squirrel (*Callosciurus erythraeus*)^R,P,†^	16.52 ± 4.87 (n = 23)	25.74 ± 7.59 (n = 5)
Masked palm civet (*Paguma larvata*)^F,†^	10.69 ± 8.42 (n = 29)	62.73 ± 15.25 (n = 5)
Chinese bamboo rat (*Rhizomys sinensis*)^F,†^	42.76 ± 20.68 (n = 29)	18.64 ± 7.58 (n = 5)
Malayan porcupine (*Hystrix brachyura*)^F,†^	10.00 ± 0.00 (n = 29)	68.06 ± 14.23 (n = 5)
Chinese muntjac (*Muntiacus reevesi*)^F,†^	10.00 ± 0.00 (n = 29)	142.62 ± 49.67 (n = 5)
Coypu (*Myocastor coypus*)^F^	5.00 ± 0.00 (n = 29)	28.70 ± 5.08 (n = 5)
Marmot (*Marmota himalayana*)^F^	15.00 ± 4.29 (n = 20)	81.37 ± 11.70 (n = 5)
Red fox (*Vulpes vulpes*)^F,†^	30.00 ± 0.00 (n = 25)	60.96 ± 21.68 (n = 5)
Mink (*Neovison vison*)^F^	10.37 ± 1.92 (n = 27)	34.62 ± 14.78 (n = 5)
Red squirrel (*Sciurus vulgaris*)^R,P,†^	16.43 ± 9.51 (n = 28)	26.04 ± 8.14 (n = 5)
Wild boar (*Sus scrofa*)^W,R,F,^*^,†^	(4.17 ± 5.77, n = 29)	319.57 ± 55.95 (n = 5)
Complex-toothed Flying Squirrel (*Trogopterus xanthipes*)^F,P,†^	5.17 ± 27.85 (n = 29)	28.11 ± 9.64 (n = 5)
**Birds**		
Collared crow (*Corvus torquatus*)^R,P^	9.14 ± 20.18 (n = 29)	54.74 ± 8.43 (n = 5)
Spotted dove (*Spilopelia chinensis*)^R,F,†^	200.00 ± 0.00 (n = 29)	7.54 ± 1.10 (n = 5)
Eurasian magpie (*Pica pica*)^R,F,P,†^	21.54 ± 28.53 (n = 13)	10.21 ± 3.56 (n = 5)
Crested myna (*Acridotheres cristatellus*)^R,P,†^	60.34 ± 20.61 (n = 29)	15.39 ± 16.23 (n = 5)
Chukar partridge (*Alectoris chukar*)^F,†^	273.68 ± 45.24 (n = 19)	6.66 ± 1.38 (n = 5)
Ring-necked Pheasant (*Phasianus colchicus*)^F,†^	80.00 ± 0.00 (n = 26)	14.80 ± 5.44 (n = 5)
Peacock (*Pavo cristatus*)^F,P,^*	15.00 ± 0.00 (n = 15)	55.63 ± 20.33 (n = 5)
Guinea fowl (*Numida meleagris*)^F^	35.00 ± 15.81 (n = 10)	12.13 ± 5.17 (n = 5)
**Reptiles**		
Beauty rat snake (*Orthriophis taeniurus*)^R,F,†^	(7.00 ± 10.90, n = 28)	22.78 ± 15.36 (n = 5)
Red large-toothed Snake (*Dinodon rufozonatum*)^R,F,†^	(7.78 ± 11.56, n = 27)	10.06 ± 4.84 (n = 5)
Many-banded krait (*Bungarus multicinctus*)^R,F,†^	(3.18 ± 3.32, n = 27)	11.24 ± 3.41 (n = 5)
Ringed water snake (*Sinonatrix annularis*)^R,P,†^	(19.00 ± 39.21, n = 29)	3.25 ± 1.24 (n = 5)
Short-tailed pit viper (*Gloydius brevicaudu*s)^R,F,†^	(5.96 ± 10.30, n = 27)	7.84 ± 1.93 (n = 5)
Chinese cobra (*Naja atra*)^R,F,†^	(59.04 ± 54.93, n = 28)	N/A
Monocled cobra (*Naja kaouthia*)^F,†^	(18.48 ± 48.50, n = 29)	20.42 ± 6.57 (n = 5)
Oriental rat snake (*Ptyas mucosa*)^F,†^	(11.76 ± 20.44, n = 29)	18.94 ± 3.21 (n = 5)
Sharp-nosed pit viper (*Deinagkistrodon acutus*)^F,†^	(3.69 ± 5.35, n = 26)	41.13 ± 16.65 (n = 5)
Siamese crocodile (*Crocodylus siamensis*)^F,^*	(2.07 ± 2.53, n = 27)	N/A
Big-eyed rat snake (*Ptyas dhumnades*)^R,F,†^	(121.10 ± 138.11, n = 29)	10.36 ± 2.09 (n = 5)
King rat snake (*Elaphe carinata*)^R,F,†^	(104.97 ± 85.07, n = 29)	N/A

Individuals sourced directly from the wild were inferred from wounds (W) and/or according to vendor responses (R). Species were sold either for food (F) and/or pets (P). The permitted species listed on the vendor’s license are labelled with a * symbol. The 31 species labelled with † are protected under the LESS. Because all reptiles and 3 mammal species (Siberian weasel (*Mustela sibirica*), Hog badger (*Arctonyx albogularis*) and Wild boar (*Sus scrofa*)) were sold by weight, vendors did not record the number of traded individuals for these species. Therefore, we include in parenthesis the average number of individuals X.X. counted on sale on his monthly market visits. Similarly, because reptiles were priced by weight (Chinese cobra (*Naja atra*): $20.71 ± 4.68 (n = 5) per kg; Siamese crocodile (*Crocodylus siamensis*): $21.01 ± 5.38 (n = 5) per kg; King rat snake (*Elaphe carinata*): $21.90 ± 5.77 (n = 5) per kg), we cannot give a price per individual, and thus indicate this as N/A in the Table. Standard deviation per species reflects variation in sales per month; complete consistency (SD = 0) implies a regular supply of individuals.

**Figure 2:**
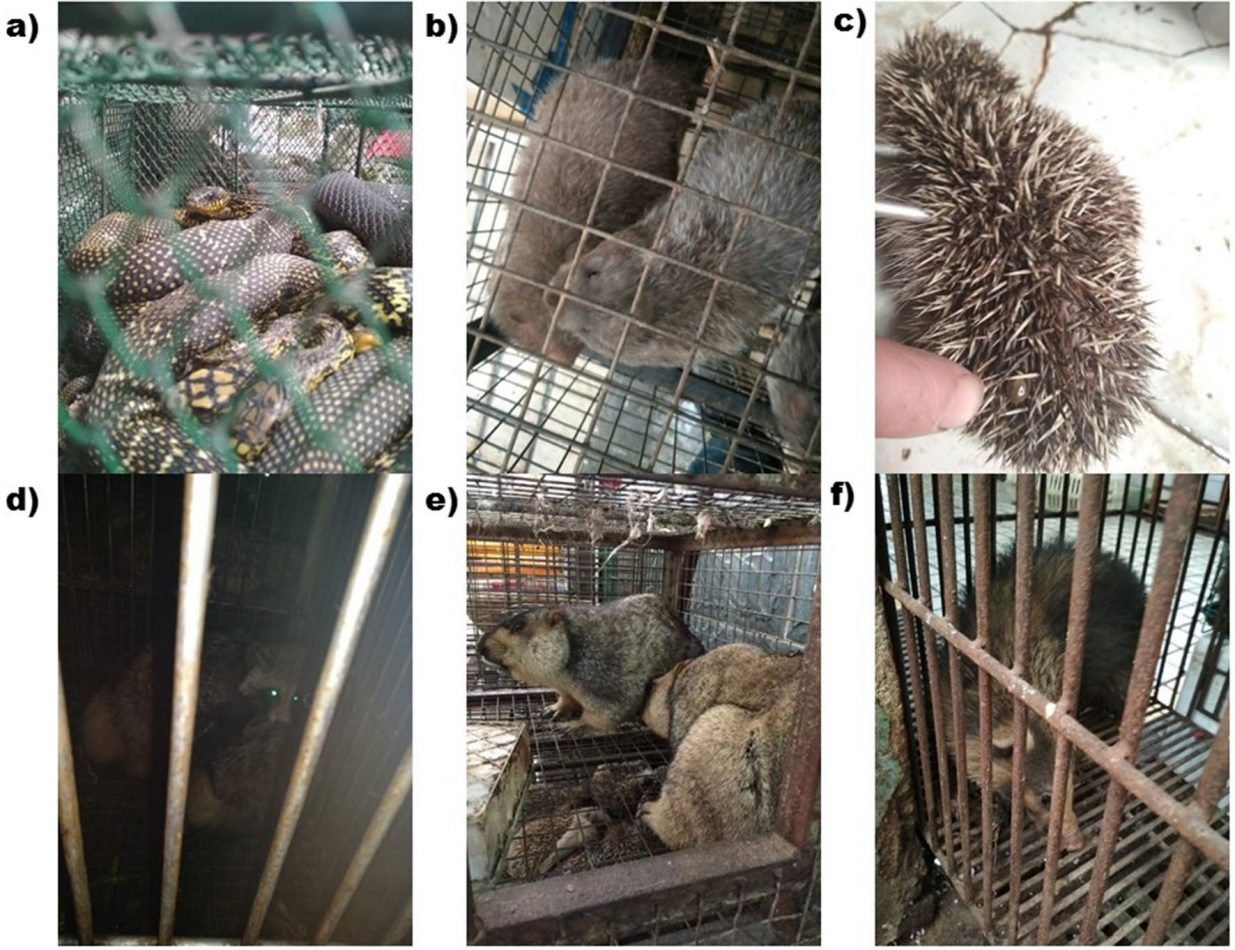
Poor welfare of animals on sale in Huanan seafood market: (**a**) King rat snake (*Elaphe carinata*), (**b**) Chinese bamboo rat (*Rhizomys sinensis*), (**c**) Amur hedgehog (*Erinaceus amurensis*) (the finger points to a tick), (**d**) Raccoon dog (*Nyctereutes procyonoides*), (**e**) Marmot (*Marmota himalayana*) (beneath the marmots is a cage containing hedgehogs), and (**f**) Hog badger (*Arctonyx albogularis*).

Almost all animals were sold alive, caged, stacked and in poor condition (Fig. 2). Most stores offered butchering services, done on site, with considerable implications for food hygiene and animal welfare. Approximately 30% of individuals from 6 mammal species inspected (labelled W in Table 1) had suffered wounds from gunshots or traps, implying illegal wild harvesting (Table 1). Thirteen of these 17 stores clearly posted the necessary permits from Wuhan Forestry Bureau allowing them to sell legitimate wild animal species (e.g., Siamese Crocodile (*Crocodylus siamensis*), Indian Peafowl (*Pavo cristatus*), Common Pheasant (*Phasianus colchicus*) and Amur hedgehog *(Erinaceus amurensis*)) for food; four shops had no such permit. Species names were given in Chinese only, with no clear taxonomic binomial designation. None of the 17 shops posted an origin certificate or quarantine certificate, so all wildlife trade was fundamentally illegal. Notably, vendors freely disclosed a variety of protected species on sale illegally in their shops, therefore they would not benefit from specifically concealing pangolin trade or the trade in any particular species, and so we are confident this list is complete (Table 1).

The most expensive wild mammal species sold for food was the marmot (*Marmota himalayana*) at over US$ 25 per kg, while raccoon dogs (*Nyctereutes procyonoides*) and badgers (*Arctonyx albogularis* and *Meles leucurus*) were priced at c. $ 15–20 per kg; hedgehogs retailed for as little as $ 2–3 each; all wild caught and intended as food. Squirrels (*Callosciurus erythraeus* and *Sciurus vulgaris*) were sold as pets for c. $ 25 each. The most expensive wild bird sold for food was the Indian peafowl (*Pavo cristatus*) at $ 56 each, while captive-bred crested myna birds (*Acridotheres cristatellus*), prized for imitating human speech, were sold as pets for c. $ 300. The Sharp-nosed pit viper (*Deinagkistrodon acutus*) was the most expensive reptile, at $ 70 per kg. For comparison, the average retail prices of pork, poultry and fish in Wuhan were $ 5.75, $ 4.25 and $ 2.32, respectively (source: Municipal Bureau of Commerce http://sw.wuhan.gov.cn/html/ztzl/sjfb/scyx/jgjczb/).

These traded species are capable of hosting a wide range of infectious zoonotic diseases or disease-baring parasites (see Supplementary Table S1 for a non-exhaustive summary of studies reporting diseases in these species in China since 2009).

## Discussion

Our findings illustrate both the range and extent of wildlife exploitation in Wuhan markets, prior to new trading bans linked to the COVID-19 outbreak, along with the poor conditions under which these animals were kept prior to sale. Circumstantially, the absence of pangolins (and bats, not typically eaten in Central China; media footage generally depicts Indonesia) from our comprehensive survey data corroborates that pangolins are unlikely implicated as spill-over hosts in the COVID-19 outbreak. This is unsurprising because live pangolin trading has largely ceased in China^13^.

We should therefore not be complacent, because the original source of COVID-19 does not seem to have been established. This is doubly important because false attribution can lead to extreme and irresponsible animal persecution. For instance, civets were killed *en masse* following the SARS-CoV outbreak^5^, and any unwarranted vilification or persecution of pangolins and bats in relation to COVID-19 would risk undermining otherwise very successful efforts to better protect and conserve wildlife in China.

Regarding our insights into broader IWT issues in Wuhan, the animals sold were relatively expensive, representing luxury food items, not cheap bushmeat (Table 1). We thus make an ethical distinction here between the subsistence consumption of bush meat in poorer nations, versus the sort of cachet attached to wild animal consumption in parts of the developed world, notably China^14^, but also Japan^15^. While c. 30% of mammals were clearly wild-caught, indicated by trapping and shooting wounds, the captive breeding of other species is commonplace in China. Raccoon dog fur farming is legal in China; however, due to a drop in fur prices, raccoon dogs are now frequently sold off in live animal markets, augmented by wild-caught individuals. Similarly, all American mink (*Neovison vison*) originated from fur farms—noting that SARS-CoV-2 has been reported in mink farms in Europe and North America^16, 17^. In contrast, the captive breeding and sale of Siberian weasels (*Mustela sibirica*), is totally illegal in China, yet they are easy to breed, and sold openly, without attracting law enforcement. Indeed, prior to COVID-19 reforms, although enforcement officers from the Wuhan Forestry Bureau issued permits to market vendors, they were broadly disinterested in what species were sold. Furthermore, although animals were required to have an origin certificate and be quarantined to ensure they did not exhibit overt disease symptoms, no clear policy was enforced on these conditions. This is important because the species that were traded are capable of hosting a wide range of infectious zoonotic diseases or disease-baring parasites (Supplementary Table S1), aside from COVID-19. These range from potentially lethal viruses, for example, rabies, SFTS, H5N1, to common bacterial infections that, nevertheless, represent a risk to human health (e.g., *Streptococcus*). Indeed, globally, wildlife is thought to be the source of at least 70% of all emerging diseases^18^.

Legislative reform is also vital to clarify unequivocally which species are considered ‘wild’ and cannot be traded legally and safely. Another problem, as encountered by the WHO report is that, retrospectively, it proved difficult to ascertain which species were on sale, even to the genus level, relying solely on the responsible market authority's official sales records and disclosures^1^. As we^19, 20^, and others^21^, have proposed previously, China’s LFSSP and LESS must be updated to apply proper binomials, and to align with recent taxonomic revisions; for instance, cobra snakes (*Nada atra*) can be farmed legally for food with permits, but wild caught species, such as water snakes and wolf snakes were also sold in Wuhan, labelled simply as ‘snakes’. Such an application of clear species names would allow for more effective prosecutions^19^. Furthermore, the WHO reports that market authorities claimed all live and frozen animals sold in the Huanan market were acquired from farms officially licensed for breeding and quarantine, and as such no illegal wildlife trade was identified^1^. In reality, however, because China has no regulatory authority regulating animal trading conducted by small-scale vendors or individuals it is impossible to make this determination^1, 21^. Similar discrepancies concerning species identification and origins afflict investigations around the world^22^.

Another important animal trade that requires attention, outside of exploitation as food, is the supply of pets, like the squirrels and crested myna birds sold in Wuhan’s market. Our previous research found annual trade volumes equivalent to c. 17,000 parrots and c. 160,000 turtles (many turtles being invasive if escaping to the wild) sold online as pets via Taobao.com between 2016–2017, in contravention of China’s WACL and/or the Animal Epidemic Prevention Law^23–25^. While not currently the vector of any major viral epidemics, it would be naive to imagine that unconventional pets do not still also pose a serious concern for public health^26^. This potential for disease is likely exacerbated by poor sanitary and welfare conditions (Fig. 2).

## Conclusion

Ultimately, changing the attitudes of consumers is crucial to reduce IWT in China. Efforts to stem the trade in charismatic species, such as elephants/ivory, rhino/horn, tiger bones, etc., have achieved modest success, and have garnered worldwide media attention and public concern^27^. Nevertheless, despite a general decrease in wildlife poaching and trafficking in China^12^, attempts to dissuade people from consuming lower-profile, but also higher-volume, species have still fallen-short. Crucially, efforts must be made to change the normative values of consumers through education, raising awareness not only for health, but also for animal welfare and global biodiversity concerns, else continued demand, despite recent national bans, may merely push suppliers into black-market and dark-web operations^23^. Our own previous investigation found that, in China, a substantial desire to purchase and/or own wildlife products as ‘prestige items’ still transcends social classes, age groups, education levels and rural versus urban residents, even though this involves breaking the law^14^. In major part this is because protective legislation has not been enforced consistently, fostering a nonchalant disregard for wildlife exploitation^23^.

President Xi Jinping has said that: *the COVID-19 outbreak is a major test of China's system and capacity for governance, and we must sum up the experience and draw a lesson from it*. In an early precautionary response to the COVID-19 crisis, on the 26th of January 2020, the Chinese government (State Administration for Market Regulation, Ministry of Agriculture and Rural Affairs and National Forestry and Grassland Administration) temporarily banned the sale of all wild animal and their products in markets, restaurants and over e-commerce, to least until the conclusion of the epidemic. Subsequently, on 24th Feb 2020, the Standing Committee of the National People’s Congress implemented a permanent ban on trading terrestrial wild (non-livestock) animals and consuming them as food. The Hubei provincial government announced on 11th April 2020 that the sale of live wild animals and poultry will be strictly prohibited as markets re-open in Wuhan. Ultimately China plans to invest c. $30 million to update Wuhan open-air markets to megamalls and inspire social capital. This is a major and commendable step, redressing previous tacit tolerance for many forms of wildlife trade in China, often already illegal under WACL and/or the CITES, which also carries a huge collateral benefit for global biodiversity and animal welfare^28, 29^. In response to the pandemic, on April 12th 2021 the WHO (co-signed by UNEP and OIE) released interim guidance for ‘Reducing public health risks associated with the sale of live animals on mammalian species in traditional food markets’^30^. Adopting these more responsible practices has the potential to save countless lives in the future.

## Supplementary Information

Supplementary Information.

## Data Availability

All datasets used in this study are included in the main text and the electronic supplementary material.
