# Supplementary material for: Animal sales from Wuhan wet markets immediately prior to the COVID-19 pandemic
Source: Sci Rep. 2021 Jun 7;11:11898. doi: 10.1038/s41598-021-91470-2 (PMC8184983; doi:10.1038/s41598-021-91470-2)
Supplement: Supplementary file 1 — Supplementary Information. [file 41598_2021_91470_MOESM1_ESM.pdf]

Animal sales from Wuhan wet markets immediately prior to the COVID-19 pandemic

Xiao Xiao, Chris Newman, Christina D Buesching, David W Macdonald, Zhao-Min Zhou

Table S1.

List of 38 species sold in Wuhan City markets from May 2017-Nov 2019, of which thirty-three have been reportedly infected with zoonotic pathogens in wild populations, markets and/or farms in China since 2009. Note: The masked palm civet (*Paguma larvata*) was the host involved in the 2003 SARS outbreak*.

**Species on sale**	Zoonotic pathogens found in wild populations	Zoonotic pathogens found in markets	Zoonotic pathogens found in farmed hosts
**Mammals**			
Raccoon dog (*Nyctereutes procyonoides*)			*Enterocytozoon bieneusi* (Yang et al. 2015), H5N1 Influenza (Qi et al. 2009), Arctic-like rabies virus (Shao et al. 2011), *Toxoplasma gondii* (Zheng et al. 2017), Carnivore amdoparvovirus (Shao et al. 2014), canine distemper virus (Zhao et al. 2010; Cheng et al. 2015), *Enterocytozoon bieneusi* (Xu et al. 2016)
Amur hedgehog (*Erinaceus amurensis*)	*Coxiella burnetii* (Gong et al. 2020), *Enterocytozoon bieneusi* (Gong et al. 2021), *Leptospira interrogans* (Ma et al. 2020a)		
Siberian weasel (*Mustela sibirica*)			
Hog badger (*Arctonyx albogularis*)			
Asian badger (*Meles leucurus*)	*Babesia* spp. (Sang et al. 2021)		
Chinese hare (*Lepus sinensis*)	*Klebsiella pneumoniae* (Du et al. 2014)		

Pallas's squirrel (*Callosciurus erythraeus*)		*Enterocytozoon bieneusi* (Deng et al. 2016), *Cryptosporidium* spp. (Chai et al. 2019),	
Masked palm civet* (*Paguma larvata*)			*Enterocytozoon bieneusi*, *Giardia duodenalis* and *Cryptosporidium* spp. (Yu et al. 2020), *Toxoplasma gondii* (Hou et al. 2016)
Chinese bamboo rat (*Rhizomys sinensis*)	*Penicillium marneffei* (Cao et al. 2011)		*Enterocytozoon bieneusi* (Wang et al. 2019), *Cryptosporidium* spp. (Wei et al. 2019; Li et al. 2020), *Giardia duodenalis* (Ma et al. 2018)
Malayan porcupine (*Hystrix brachyura*)			
Chinese muntjac (*Muntiacus reevesi*)			
Coypu (*Myocastor coypus*)			*Giardia duodenalis* (Cui et al. 2021), *Enterocytozoon bieneusi* (Yu et al. 2020)
Marmot (*Marmota himalayana*)	*Yersinia pestis* (Xu et al. 2018), *Enterocytozoon bieneusi* (Xu et al. 2020)		
Red fox (*Vulpes vulpes*)			*Enterocytozoon bieneusi* (Yang et al. 2015; Ma et al. 2020b),
Mink (*Neovison vison*)			*Enterocytozoon bieneusi* (Zhang et al. 2018), Newcastle disease virus (Zhao et al. 2014), severe pseudorabies virus (Liu et al. 2017), *Circovirus* (Lian et al. 2014)
Red squirrel (*Sciurus vulgaris*)		*Cryptosporidium* spp., *Enterocytozoon bieneusi* (Deng et al. 2020)	
Wild boar (*Sus scrofa*)			*Clostridium perfringens* (Li et al. 2017), *Enterocytozoon bieneusi*

			and *Cryptosporidium* spp. (Feng et al. 2020), *Toxoplasma gondii* (Bai et al. 2017)
Complex-toothed Flying Squirrel (*Trogopterus xanthipes*)			*Leptospira*, *Blastocystis* (Xiao et al. 2019a)
**Birds**			
Collared crow (*Corvus torquatus*)			
Spotted dove (*Spilopelia chinensis*)	Newcastle disease virus (Duan et al. 2014; Xiang et al. 2017)		
Eurasian magpie (*Pica pica*)		*Toxoplasma gondii* (Chen et al. 2015)	
Crested myna (*Acridotheres cristatellus*)		*Enterocytozoon bieneusi* (Deng et al. 2019), *Cryptosporidium* spp. (Li et al. 2016)	
Chukar partridge (*Alectoris chukar*)			Duck Influenza Virus Subtype H9N2 (Zhu et al. 2018)
Ring-necked Pheasant (*Phasianus colchicus*)			Avian coronaviruses (Han et al. 2020)
Peacock (*Pavo cristatus*)			H5N6 avian influenza virus (Li et al. 2019), *Cryptosporidium* spp., *Enterocytozoon bieneusi* (Feng et al. 2016)
Guinea fowl (*Numida meleagris*)			Fowl adenoviruses (Li et al. 2019), H7N9 virus (Fan et al. 2019)
**Reptiles**			
Beauty rat snake (*Orthriophis taeniurus*)	*Spirometra* spp. (Xiao et al. 2019b)		
Red large-toothed Snake (*Dinodon rufozonatum*)			*Ophidiomyces ophiodiicola* (Sun et al. 2021)
Many-banded krait (*Bungarus multicinctus*)	*Spirometra* spp. (Xiao et al. 2019b)		
Ringed water snake (*Sinonatrix annularis*)	*Spirometra* spp. (Xiao et al. 2019b)		
Short-tailed pit viper	*Spirometra* spp., *Hepatozoon* spp.		

(*Gloydius brevicaudus*)	and *Cryptosporidium* spp. (Xiao et al. 2019b)		
Chinese cobra (*Naja atra*)		*Spirometra erinaceieuropaei* (Wang et al. 2011)	*Cryptosporidium* spp. (Xiao et al. 2019b)
Monocled cobra (*Naja kaouthia*)			*Cryptosporidium* spp. (Xiao et al. 2019b)
Oriental rat snake (*Ptyas mucosa*)		*Spirometra erinaceieuropaei* (Wang et al. 2011)	*Cryptosporidium* spp. (Xiao et al. 2019b)
Sharp-nosed pit viper (*Deinagkistrodon acutus*)			*Spirometra* spp. and *Cryptosporidium* spp. (Xiao et al. 2019b)
Siamese crocodile (*Crocodylus siamensis*)			*Aeromonas* spp. (Pu et al. 2019), tilapia parvovirus (Du et al. 2019)
Big-eyed rat snake (*Ptyas dhumnades*)	*Cryptosporidium* spp. (Xiao et al. 2019b)		
King rat snake (*Elaphe carinata*)			*Cryptosporidium* spp. (Xiao et al. 2019b)

References:

Bai, M. J., Zou, Y., Elsheikha, H. M., Ma, J. G., Zheng, W. B., Zhao, Q., ... & Zhu, X. Q. (2017). *Toxoplasma gondii* infection in farmed wild boars (*Sus scrofa*) in three cities of Northeast China. *Foodborne pathogens and disease*, *14*(7), 379-385.

Cao, C., Liang, L., Wang, W., Luo, H., Huang, S., Liu, D., ... & Fisher, M. C. (2011). Common reservoirs for *Penicillium marneffei* infection in humans and rodents, China. *Emerging infectious diseases*, *17*(2), 209.

Chai, Y., Deng, L., Liu, H., Yao, J., Zhong, Z., Xiang, L., ... & Hu, Y. (2019). First detection of *Cryptosporidium* spp. in red-bellied tree squirrels (*Callosciurus erythraeus*) in China. *Parasite*, *26*.

Chen, R., Lin, X., Hu, L., Chen, X., Tang, Y.,Zhang, J., …& Huang, C. (2015). Genetic characterization of *Toxoplasma gondii* from zoo wildlife and pet birds in Fujian, China. *Iranian journal of parasitology*, *10*(4), 663.

Cheng, Y., Wang, J., Zhang, M., Zhao, J., Shao, X., Ma, Z., ... & Wu, H. (2015). Isolation and sequence analysis of a canine distemper virus from a raccoon dog in Jilin Province, China. *Virus genes*, *51*(2), 298-301.

Cui, Z., Wang, D., Wang, W., Zhang, Y., Jing, B., Xu, C., ... & Zhang, L. (2021). Occurrence and Multi-Locus Analysis of *Giardia duodenalis* in Coypus (*Myocastor coypus*) in China. *Pathogens*, *10*(2), 179.

Deng, L., Chai, Y., Luo, R., Yang, L., Yao, J., Zhong, Z., ... & Zhou, Z. (2020). Occurrence and genetic characteristics of *Cryptosporidium* spp. and *Enterocytozoon bieneusi* in pet red squirrels (*Sciurus vulgaris*) in China. *Scientific Reports*, *10*(1), 1-10.

Deng, L., Li, W., Yu, X., Gong, C., Liu, X., Zhong, Z., ... & Chen, H. (2016). First report of the human-pathogenic *Enterocytozoon bieneusi* from red-bellied tree squirrels (*Callosciurus erythraeus*) in Sichuan, China. *PLoS One*, *11*(9).

Deng, L., Yue, C. J., Chai, Y. J., Wang, W. Y., Su, X. Y., Zhou, Z. Y., ... & Cao, S. Z. (2019). New genotypes and molecular characterization of *Enterocytozoon bieneusi* in pet birds in Southwestern China. *International Journal for Parasitology: Parasites and Wildlife*, *10*, 164-169.

Du, J., Wang, W., Chan, J. F. W., Wang, G., Huang, Y., Yi, Y., ... & Yin, F. (2019). Identification of a novel ichthyic parvovirus in marine species in Hainan island, China. *Frontiers in microbiology*, *10*, 2815.

Du, Y., Luo, J., Wang, C., Wen, Q., Duan, M., Zhang, H., & He, H. (2014). Detection of drug-resistant *Klebsiella pneumoniae* in Chinese hares (*Lepus sinensis*). *Journal of Wildlife Diseases*, *50*(1), 109-112.

Duan, X., Zhang, P., Ma, J., Chen, S., Hao, H., Liu, H., ... & Yang, Z. (2014). Characterization of genotype IX Newcastle disease virus strains isolated from wild birds in the northern Qinling Mountains, China. *Virus genes*, *48*(1), 48-55.

Feng, S. Y., Chang, H., Luo, J., Huang, J. J., & He, H. X. (2019). First report of *Enterocytozoon bieneusi* and *Cryptosporidium* spp. in peafowl (*Pavo cristatus*) in China. *International Journal for Parasitology: Parasites and Wildlife*, *9*, 1-6.

Feng, S., Jia, T., Huang, J., Fan, Y., Chang, H., Han, S., ... & He, H. (2020). Identification of *Enterocytozoon bieneusi* and *Cryptosporidium* spp. in farmed wild boars (*Sus scrofa*) in Beijing, China. *Infection, Genetics and Evolution*, *80*, 104231.

Gong, X. Q., Xiao, X., Liu, J. W., Han, H. J., Qin, X. R., Lei, S. C., & Yu, X. J. (2020). Occurrence and Genotyping of Coxiella burnetii in Hedgehogs in China. *Vector-Borne and Zoonotic Diseases*, *20*(8), 580-585.

Han, Z., Liwen, X., Ren, M., Sheng, J., Ma, T., Sun, J., ... & Liu, S. (2020). Genetic, antigenic and pathogenic characterization of avian coronaviruses isolated from pheasants (*Phasianus colchicus*) in China. *Veterinary Microbiology*, *240*, 108513.

Hou, G. Y., Zhao, J. M., Zhou, H. L., & Rong, G. (2016). Seroprevalence and genetic characterization of *Toxoplasma gondii* in masked palm civet (*Paguma larvata*) in Hainan province, tropical China. *Acta tropica*, *162*, 103-106.

Li, F., Zhang, Z., Hu, S., Zhao, W., Zhao, J., Kváč, M., ... & Xiao, L. (2020). Common occurrence of divergent *Cryptosporidium* species and *Cryptosporidium parvum* subtypes in farmed bamboo rats (*Rhizomys sinensis*). *Parasites & vectors*, *13*, 1-8.

Li, M., Feng, S., Lv, S., Luo, J., Guo, J., Sun, J., & He, H. (2019). Highly pathogenic H5N6 avian influenza virus outbreak in *Pavo cristatus* in Jiangxi Province, China. *Emerging Microbes & Infections*, *8*(1), 377-380.

Li, M., Zhang, X., Zhu, L., Wang, H., Zhao, N., Luo, J., ... & Zhang, B. (2017). Identification, Isolation, and Phylogenetic Analysis of *Clostridium perfringens* Type A and Type C from Wild Boar (*Sus scrofa*) in the People's Republic of China. *Journal of Wildlife Diseases*, *53*(3), 642-648.

Li, Q., Li, L., Tao, W., Jiang, Y., Wan, Q., Lin, Y., & Li, W. (2016). Molecular investigation of *Cryptosporidium* in small caged pets in northeast China: host specificity and zoonotic implications. *Parasitology Research*, *115*(7), 2905-2911.

Lian, H., Liu, Y., Li, N., Wang, Y., Zhang, S., & Hu, R. (2014). Novel circovirus from mink, China. *Emerging infectious diseases*, *20*(9), 1548.

Liu, H., Li, X. T., Hu, B., Deng, X. Y., Zhang, L., Lian, S. Z., ... & Yan, X. J. (2017). Outbreak of severe pseudorabies virus infection in pig-offal-fed farmed mink in Liaoning Province, China. *Archives of virology*, *162*(3), 863-866.

Ma, X. J., Gong, X. Q., Xiao, X., Liu, J. W., Han, H. J., Qin, X. R., ... & Yu, X. J. (2020a). Detection of *Leptospira interrogans* in hedgehogs from Central China. *Vector-Borne and Zoonotic Diseases*, *20*(6), 427-431.

Ma, X., Wang, Y., Zhang, H. J., Wu, H. X., & Zhao, G. H. (2018). First report of *Giardia duodenalis* infection in bamboo rats. *Parasites & Vectors*, *11*(1), 520.

Ma, Y. Y., Zou, Y., Ma, Y. T., Nie, L. B., Xie, S. C., Cong, W., ... & Zhu, X. Q. (2020b). Molecular detection and genotype distribution of *Enterocytozoon bieneusi* in farmed silver foxes (*Vulpes vulpes*) and arctic foxes (*Vulpes lagopus*) in Shandong Province, eastern China. *Parasitology Research*, *119*(1), 321-326.

Pu, W., Guo, G., Yang, N., Li, Q., Yin, F., Wang, P., ... & Zeng, J. (2019). Three species of Aeromonas (*A. dhakensis*, *A. hydrophila* and *A. jandaei*) isolated from freshwater crocodiles (*Crocodylus siamensis*) with pneumonia and septicemia. *Letters in Applied Microbiology*, *68*(3), 212-218.

Qi, X., Li, X., Rider, P., Fan, W., Gu, H., Xu, L., Yang, Y., Lu, S., Wang, H., & Liu, F. (2009). Molecular characterization of highly pathogenic H5N1 avian influenza A viruses isolated from raccoon dogs in China. *PloS one*, *4*(3), e4682. https://doi.org/10.1371/journal.pone.0004682

Sang, C., Yang, Y., Dong, Q., Xu, B., Liu, G., Hornok, S., ... & Hazihan, W. (2021). Molecular survey of Babesia spp. in red foxes (*Vulpes vulpes*), Asian badgers (*Meles leucurus*) and their ticks in China. *Ticks and Tick-borne Diseases*, 101710.

Shao, X. Q., Wen, Y. J., Ba, H. X., Zhang, X. T., Yue, Z. G., Wang, K. J., ... & Yang, F. H. (2014). Novel amdoparvovirus infecting farmed raccoon dogs and arctic foxes. *Emerging Infectious Diseases, 20*(12), 2085.

Shao, X. Q., Yan, X. J., Luo, G. L., Zhang, H. L., Chai, X. L., Wang, F. X., ... & Zhang, Y. Z. (2011). Genetic evidence for domestic raccoon dog rabies caused by Arctic-like rabies virus in Inner Mongolia, China. *Epidemiology & Infection*, *139*(4), 629-635.

Sun, P. L., Yang, C. K., Li, W. T., Lai, W. Y., Fan, Y. C., Huang, H. C., & Yu, P. H. (2021). Infection with *Nannizziopsis guarroi* and *Ophidiomyces ophiodiicola* in reptiles in Taiwan. *Transboundary and Emerging Diseases*.

Wang, F., Zhou, L., Gong, S., Deng, Y., Zou, J., Wu, J., ... & Hou, F. (2011). Severe infection of wild-caught snakes with *Spirometra erinaceieuropaei* from food markets in Guangzhou, China involves a risk for zoonotic sparganosis. *Journal of Parasitology*, *97*(1), 170-171.

Wang, H., Liu, Q., Jiang, X., Zhang, Y., Zhao, A., Cui, Z., ... & Zhang, L. (2019). Dominance of zoonotic genotype D of *Enterocytozoon bieneusi* in bamboo rats (*Rhizomys sinensis*). *Infection, Genetics and Evolution*, *73*, 113-118.

Wei, Z., Liu, Q., Zhao, W., Jiang, X., Zhang, Y., Zhao, A., ... & Qi, M. (2019). Prevalence and diversity of *Cryptosporidium* spp. in bamboo rats (*Rhizomys sinensis*) in South Central China. *International Journal for Parasitology: Parasites and Wildlife*, *9*, 312-316.

Xiang, B., Han, L., Gao, P., You, R., Wang, F., Xiao, J., ... & Ren, T. (2017). Spillover of Newcastle disease viruses from poultry to wild birds in Guangdong province, southern China. *Infection, Genetics and Evolution*, *55*, 199-204.

Xiao, X., Qi, R., Han, H. J., Liu, J. W., Qin, X. R., Fang, L. Z., ... & Yu, X. J. (2019b). Molecular identification and phylogenetic analysis of *Cryptosporidium*, *Hepatozoon* and *Spirometra* in snakes from central China. *International Journal for Parasitology: Parasites and Wildlife*, *10*, 274-280.

Xiao, X., Zhou, S. H., Jiang, N., Tian, D. Z., Zhou, Z. M., Zhang, M., ... & Gao, Q. H. (2019a). First record of *Leptospira* and *Blastocystis* infections in captive flying squirrels (*Trogopterus xanthipes*) from Enshi County, China. *Acta Tropica*, *197*, 105065.

Xu, C., Ma, X., Zhang, H., Zhang, X. X., Zhao, J. P., Ba, H. X., ... & Zhao, Q. (2016). Prevalence, risk factors and molecular characterization of *Enterocytozoon bieneusi* in raccoon dogs (*Nyctereutes procyonoides*) in five provinces of Northern China. *Acta tropica*, *161*, 68-72.

Xu, J., Wang, X., Jing, H., Cao, S., Zhang, X., Jiang, Y., ... & Shen, Y. (2020). Identification and genotyping of *Enterocytozoon bieneusi* in wild Himalayan marmots (*Marmota himalayana*) and Alashan ground squirrels (*Spermophilus alashanicus*) in the Qinghai-Tibetan Plateau area (QTPA) of Gansu Province, China. *Parasites & vectors*, *13*(1), 1-8.

Xu, X., Cui, Y., Xin, Y., Yang, X., Zhang, Q., Jin, Y., ... & Jin, J. (2018). Genetic diversity and spatial-temporal distribution of *Yersinia pestis* in Qinghai Plateau, China. *PLoS Neglected Tropical Diseases*, *12*(6), e0006579.

Yang, Y., Lin, Y., Li, Q., Zhang, S., Tao, W., Wan, Q., ... & Li, W. (2015). Widespread presence of human-pathogenic *Enterocytozoon bieneusi* genotype D in farmed foxes (*Vulpes vulpes*) and raccoon dogs (*Nyctereutes procyonoides*) in China: first identification and zoonotic concern. *Parasitology Research*, *114*(11), 4341-4348.

Yu, F., Cao, Y., Wang, H., Liu, Q., Zhao, A., Qi, M., & Zhang, L. (2020). Host-adaptation of the rare *Enterocytozoon bieneusi* genotype CHN4 in *Myocastor coypus* (Rodentia: Echimyidae) in China. *Parasites & Vectors*, *13*(1), 1-8.

Yu, Z., Wen, X., Huang, X., Yang, R., Guo, Y., Feng, Y., ... & Li, N. (2020). Molecular characterization and zoonotic potential of *Enterocytozoon bieneusi*, *Giardia duodenalis* and *Cryptosporidium* sp. in farmed masked palm civets (*Paguma larvata*) in southern China. *Parasites & Vectors*, *13*(1), 1-10.

Zhang, X. X., Jiang, R. L., Ma, J. G., Xu, C., Zhao, Q., Hou, G., & Liu, G. H. (2018). *Enterocytozoon bieneusi* in minks (N*eovison vison*) in Northern China: a public health concern. *Frontiers in Microbiology*, *9*, 1221.

Zhao, J. J., Yan, X. J., Chai, X. L., Martella, V., Luo, G. L., Zhang, H. L., ... & Cheng, S. P. (2010). Phylogenetic analysis of the haemagglutinin gene of canine distemper virus strains detected from breeding foxes, raccoon dogs and minks in China. *Veterinary microbiology*, *140*(1-2), 34-42.

Zhao, P., Sun, L., Sun, X., Li, S., Zhang, W., Pulscher, L. A., ... & Xing, M. (2017). Newcastle disease virus from domestic mink, China, 2014. *Veterinary microbiology*, *198*, 104-107.

Zheng, W. B., Cong, W., Hou, J., Ma, J. G., Zhang, X. X., Zhu, X. Q., ... & Zhou, D. H. (2017). Seroprevalence and risk factors of *Toxoplasma gondii* infection in farmed raccoon dogs (*Nyctereutes procyonoides*) in China. *Vector-Borne and Zoonotic Diseases*, *17*(3), 209-212.

Zhu, Y. C., Zhang, B., Sun, Z. H., Wang, X. J., Fan, X. H., Gao, L. X., ... & Zhang, Z. F. (2018). Replication and pathology of duck influenza virus subtype H9N2 in Chukar. *Biomedical and environmental sciences*, *31*(4), 306-310.
